# Preventing elder maltreatment: Identification of high risk factors from LTC administrative data

**DOI:** 10.1093/geroni/igab046.3522

**Published:** 2021-12-17

**Authors:** Shiau-Fang Chao, Chen-Wei Hsiang, Kuan-Ming Chen, Ya-Mei Chen, Ji-Lung Hsieh, Yu-Hsuan Chou, Ming-Jen Lin, Shih-Cyuan Wu

**Affiliations:** 1 National Taiwan University, Taipei City, Taiwan (Republic of China); 2 National Taiwan University, National Taiwan University, Taipei, Taiwan (Republic of China); 3 National Bureau of Economic Research, CAMBRIDGE, Massachusetts, United States; 4 National Taiwan University, Taipei, Taipei, Taiwan (Republic of China)

## Abstract

Elder maltreatment is a serious problem endangering physical, emotional, and material well-being of older persons, especially those with physical and cognitive impairment. However, detecting the incident of elder maltreatment is difficult and its prevalence has been seriously underestimated. This study explores how LTC use relates to elder maltreatment report, using government LTC service records in Taiwan. A total of 88,633 reported cases in adult protection system in 2019 were merged with 443,952 valid cases in LTC service system. Descriptive statistics were firstly performed to examine the proportion and characteristics of repeated cases in both systems. Linear probability modeling was then used for analyses. 1. In 2019, 3,413 elder maltreatment clients can be identified in LTC service system, accounting for 27.3% of the elder maltreatment cases. 2. Older persons who used LTC service first and being reported as elder maltreatment cases later had a higher prevalence of being discovered by social workers and care attendants. 3. These group of clients also had higher proportion of being reported as neglected by others, abandonment, and self-neglected. 4. Characteristics in LTC service system, such as being older, low severity of disability, high cognitive impairments, low income status, and with a LTC service use record, were related to high probability of being detected with elder maltreatment problems. Characteristics in LTC service system could be effective indicators in discovering potentially abusive situations of disabled older persons. Training and education are essential for LTC service providers to enhance their literacy and ability of assessing elder maltreatment.

